# ‘I try not to bother the residents too much’ – the use of capillary blood glucose measurements in nursing homes

**DOI:** 10.1186/s12912-016-0129-7

**Published:** 2016-02-04

**Authors:** Lillan Mo Andreassen, Anne Gerd Granas, Una Ørvim Sølvik, Reidun Lisbet Skeide Kjome

**Affiliations:** Department of Global Public Health and Primary Care, University of Bergen, Bergen, Norway; Department of Life Sciences and Health, Oslo and Akershus University College for Applied Sciences, Oslo, Norway; Noklus, Norwegian Quality Improvement of Primary Care Laboratories, Bergen, Norway; Department of Global Public Health and Primary Care/Centre for Pharmacy, University of Bergen, Bergen, Norway

**Keywords:** Diabetes mellitus, Capillary blood glucose measurements, Nursing homes, Healthcare professionals, Chronic disease management, Clinical guidelines, Nursing practice

## Abstract

**Background:**

Capillary blood glucose measurements are regularly used for nursing home residents with diabetes. The usefulness of these measurements relies on clear indications for use, correct measurement techniques, proper documentation and clinical use of the resulting blood glucose values. The use of a regular, invasive procedure may also entail additional challenges in a population of older, multimorbid patients who often suffer from cognitive impairment or dementia. The aim of this study was to explore the perspectives of physicians, registered nurses and auxiliary nurses on the use, usefulness and potential challenges of using capillary blood glucose measurements in nursing homes, and the procedures for doing so.

**Methods:**

This was a qualitative study that used three profession-specific focus group interviews. Interviews were transcribed in modified verbatim form and analysed in accordance with Malterud’s principles of systematic text condensation. Five physicians, four registered nurses and three auxiliary nurses participated in the focus groups.

**Results:**

All professional groups regarded capillary blood glucose measurements as a necessity in the management of diabetes, the physicians to ensure that the treatment is appropriate, and the nurses to be certain and assured about their caring decisions. Strict glycaemic control and excessive measurements were avoided in order to promote the well-being and safety of the residents. Sufficient knowledge of diabetes symptoms, equivalent practices for glucose measurement, and unambiguous documentation and communication of results were determined to be most helpful. However, all professional groups seldom involved the residents in managing their own measurements and stated that guidelines and training had been inconsistent or lacking.

**Conclusion:**

Inadequate procedures and training in diabetes care may compromise the rationale for capillary blood glucose measurements in nursing homes, and hence the residents’ safety. These concerns should be addressed together with the possibility of involving and empowering residents by exploring their ability and wish to manage their own disease.

## Background

Nursing home residents with diabetes are medically complex, with a high level of disability, many complications and medicines [1–3]. Feeding or swallowing difficulties, acute illnesses or infections, or use of insulin and other hypoglycaemic medicines can cause detrimental fluctuations in blood glucose levels. Symptoms are sometimes confused with other age-related changes or are less marked compared to symptoms in younger adults [4, 5]. Regular capillary blood glucose measurements (CBGM) are therefore recommended for these patients [6–8].

For CBGM to be useful, it requires a clear purpose, correct sampling and good analytical performance of the device used, as well as appropriate documentation, interpretation and use of the result. However, studies have reported findings such as: that CBGM is not always performed according to individual needs [9–11]; pathogen transmission due to incorrect sampling [12–14]; insufficient blood glucose logs [15, 16]; uncertainty concerning physician involvement [15] and actual use of test results [17]; lack of procedures and inconsistent instructions [15, 18, 19]. In addition, training and guidance about symptoms requiring additional measurements are not always adequate [19, 20].

Incorrect sampling or unnecessary use of CBGM puts residents at risk, adds costs and is associated with a higher burden of depression, distress and worries [21, 22]. In Norway, CBGM is the standard method for day-to-day monitoring of diabetes in nursing homes, and three quarters of nursing home residents with diabetes regularly receive CBGM [23]. Clinical procedures recommend that an individual plan for CBGM should be decided in collaboration between the physician, nursing staff and the resident [7]. However, two recent focus group studies among nurses in Norwegian nursing homes, revealed deficiencies in work procedures for diabetes care, differences of opinions about who should decide the frequency of CBGM, and poor inter-professional collaboration [24, 25].

This study is part of LMA’s PhD project on diabetes in nursing homes. In a previous study we investigated diabetes therapy and glycaemic control. One of our findings was that 60 % of the nursing home residents had at least one CBGM reading that was consistent with risk of hypoglycaemia [23]. Together with observations during data collection indicating that CBGM was an area of concern to the healthcare professionals, this led us to question whether the practices relating to CBGM were adequate to ensure the residents’ safety and well-being. This study therefore seeks to gain a better understanding of CBGM practices by exploring the perspectives of physicians, registered nurses and auxiliary nurses on the use, usefulness and potential challenges of using CBGM in nursing homes, and the procedures for doing so.

## Methods

### Design of the study

We conducted profession-specific focus group interviews with physicians, registered nurses and auxiliary nurses employed in nursing homes. Through a series of open ended-questions, focus groups interviews use the interaction between the participants to investigate their common experiences, priorities and attitudes [26].

### Participants

Three focus groups with a total of 12 participants were held in June and September 2014. Nurses were recruited in May and June 2014 through nursing home managers at two different, but geographically adjacent nursing homes. The managers received written information about the study and predetermined dates for the interviews, which they distributed to eligible employees. They then informed us how many of each professional group had agreed to participate. Physicians were recruited by visiting continuing professional education meetings for nursing home physicians in June and September 2014.

In Norway, registered nurses have a bachelor’s degree in nursing, which requires a minimum of three years education and practical training at a university college. Auxiliary nurses are licensed practical nurses, who have two years of vocational education followed by a two-year apprenticeship. Auxiliary nurses work under the guidance of registered nurses. They are also known as healthcare assistants or nursing assistants. The nursing home physicians are either full-time employed or part-time contracted general practitioners working at a nursing home once or twice a week.

For all professional groups, men and women with a licence to practice and with work experience from a nursing home were invited. No limits were set as regards the length of work experience, but it was specified in the invitation that the participants should have experience of performing or managing CBGM in a nursing home setting.

Three auxiliary nurses (AN) and four registered nurses (RN), all women from two geographically adjacent nursing homes, participated in two separate focus groups. Another two auxiliary nurses were originally recruited, but failed to show up. Five physicians (P) participated in the final focus group, two men and three women. They were employed at different nursing homes, but knew each other from regular continuing professional education meetings.

### Setting

The focus group interviews with the nurses were conducted in a meeting room at one of the nursing homes after the participants’ working hours. The focus group interview with the physicians was conducted after a continuing professional education meeting, in an adjacent meeting room. Each interview lasted between 60 and 75 min and was audiotaped. Researcher LMA moderated all interviews, and UØS, GBBK and RLSK took turns as co-moderators. The interview guide was semi-structured with open-ended questions about experience of the use, documentation, interpretation and consequences of CBGM in a nursing home setting, as well as potential challenges for patients or personnel (Table 1). Participants received a complimentary gift voucher worth EUR 45.

**Table 1:** Themes and key questions serving as guidance during data collection

Reasons for CBGM	
	Nurses Tell us about what triggered measurement the last time you performed CBGM.
	Physicians Tell us about your approach for deciding if and when a resident with diabetes should receive CBGM
Quality, documentation and communication of CBGM readings	
	Nurses/Physicians Please describe what happens with the CBGM readings at your place of work
Acute events	
	Nurses Tell us about an episode where you experienced either a high or a low blood glucose reading in a resident with diabetes.
	Physicians Tell us about an episode where you experienced or were called upon for either a high or a low blood glucose reading in a resident with diabetes.
Education and training	
	Nurses Tell us about the training you have received on diabetes care and CBGM.
	Physicians Please describe what type of training or education initiatives that exist/are given at your place of work on diabetes care and CBGM.

In this table “Nurses” refer to both registered nurses and auxiliary nurses; the key questions were identical for these two professional groups

CBGM = capillary blood glucose measurements

### Analysis

All interviews were transcribed in modified verbatim form by LMA. The analysis followed the principles for systematic text condensation (STC) [27]. We did not use a theoretical framework for this study, as we emphasised a more descriptive approach. Even though a theoretical framework can support STC analysis, STC is also often used without additional theory. STC is founded on phenomenology and the theory that knowledge is constructed through joint understandings of the world. STC offers a pragmatic, but systematic approach that safeguards transparency, inter-subjectivity, reflexivity and the feasibility of the study [27].

STC is a four-step process, defined by Malterud as 1) from chaos to themes – obtaining an overview of initial themes; 2) from themes to codes – identifying and sorting units of meaning; 3) from code to meaning – condensation of the meaning units into an abstracted text; and 4) from condensation to descriptions and concepts – synthesising the contents of the condensates. In detail, all authors first read all the transcripts in order to identify initial themes, which were used as starting categories for coding. The four themes agreed on were: needs and benefits of CBGM; glycaemic control – target values, purpose and challenges; professional knowledge, clinical skills and understanding of roles; and documentation and interaction. Secondly, LMA analysed the material iteratively based on these initial themes, searching for units of meaning. Related units were grouped under the same code heading, which was developed from the initial theme and adjusted during analysis. A fifth code group emerged during analysis: the patient perspective. In the third step, all the authors came together to sort the content of the five code groups into subgroups. LMA then condensed and abstracted the content of each subgroup into an artificial quote. In the final step, the artificial quotes within each code group were transformed by LMA into an analytical text accompanied by authentic illustrative quotes. Comparing these analytical texts to the original material, all authors searched for additional perspectives and, lastly, defined the following categories for presenting the results: 1) Premises for CBGM, 2) Professional competence and understanding of roles, 3) Record keeping. The analysis process was facilitated by the text analysis software NVivo version 10 (QSR International Pty Ltd).

### Literature search

A systematic literature search was conducted to obtain an overview of existing literature on capillary blood glucose measurements in nursing homes. The databases PubMed (EMBASE), CINAHL and MEDLINE (Ovid) were searched for relevant publications. The following search terms were used in different combinations: diabetes mellitus; nursing homes; homes for the aged; long-term care; health knowledge, attitudes, practice; attitude of health personnel; employee attitudes; professional practice; quality of health care; blood glucose; blood glucose measurement; blood glucose monitoring.

### Ethical considerations

The Norwegian Social Science Data Services (NSD) is the advisory body on privacy and research ethics for research involving healthcare professionals. NSD was consulted, but, since no personal data were registered or stored as part of the data collection, the study was not subject to notification. However, the study complied with ethical principles for research in order to protect the privacy of the participants. Specifically, the names of the participants or their workplace were not linked to the interview data, and audio recordings of the interviews were deleted once the transcripts were completed. No individual participant or nursing home could be identified in the transcripts or the finalised study results. Furthermore, all participants were given an information leaflet prior to the focus group interviews. It described the study aims, what participation entailed and the storage of data, and stated that participants could withdraw their consent at any time up until after participation without providing any reason. The leaflet also stressed the importance of professional confidentiality, reminding the participants not to identify names of patients, their families or colleagues during the interviews. This information was repeated before the interviews. Volunteering for and participation in the focus group interviews was understood as entailing consent.

## Results

### Premises for CBGM

#### Frequency and benefit of measurements

All groups expressed the view that measurements should be kept to a minimum in order to ease the strain of blood sampling (finger pricking) on the residents. The participants explained that most residents had established a relaxed and consistent CBGM regime, based on drug treatment and previous recordings of glucose levels. Physicians and registered nurses stressed the HbA1c value as central when deciding on the frequency, a decision that was made jointly according to the nurses.

*‘It varies a lot depending on [the resident’s] condition and treatment target. I try not to bother the residents too much, you know. Not to bother them more than necessary to achieve whatever treatment target I’ve set.’ P3.*

The registered nurses emphasised that a change in the resident’s situation, such as an infection, decreased food intake or exhibiting unusual symptoms, usually led them to perform more frequent measurements for a period. Both groups of nurses regarded CBGM as an easy and accessible way of confirming or disproving that a change in the residents’ cognitive or physical behaviour was due to fluctuations in their blood glucose. They trusted the readings from the CBGM devices, as the nursing homes were enrolled in an external quality assurance programme.

*‘Well, in any case, if a resident with diabetes falls ill in any way whatsoever, our first thought is, okay, we should at least check the blood sugar level, to rule it out, you know. Even if we suspect that it may be due to something completely different, we always check it, because it is such an easy and quick thing to do.’ RN2.*

All participants, but especially the physicians, regarded the measurements as essential for following up and adjusting diabetes treatment, but they admitted that they were most useful for residents with unstable blood glucose levels, or for residents in need of rapid-acting insulin.

#### Avoiding discomfort

The physicians stressed that maintaining quality of life for the residents and avoiding hypoglycaemia were the main aims when deciding the level of glycaemic control. All groups perceived the risk of long-term complications as low due to short remaining life expectancy for most residents. Hence the blood glucose levels were permitted to lie around 10 mmol/l. In their experience, this did not result in discomfort for the residents, and the registered nurses stated that a higher rather than lower blood glucose level made them feel safer as well.

*‘I’m used to them being a bit liberal, that around 10 [mmol/l] is appropriate for older persons, since they do not have that risk of long-term complications, if they’re ninety years old, you know? (…) It is safer and the residents feel fine, so if they’re in good shape and all that… But, otherwise, somewhere between 5 and 10 [mmol/l].’ RN2.*

The nurses explained that most residents achieved better glycaemic control after admission to the nursing home, probably due to regular meals and physical activity. They sometimes worried about the residents’ nocturnal blood glucose, due to the long time that elapsed between the evening meal (~7 p.m.) and breakfast (~9 a.m.). In contrast, all groups said that treats from visiting relatives often explained deviant CBGM results. However, they were ambivalent about food restrictions or preventing residents from eating what they wanted. Especially the physicians were sceptical about diets, as different-looking food made some residents feel insecure.

*‘We do not know what they eat at any given time. The wife shows up with grapes and chocolate and sugary yoghurts, and you know. That’s a bit of a challenge, to be honest.’ AN1.*

*‘In residents with dementia, I often observe that when they’re given different-looking food at mealtimes, they feel insecure and start wondering what’s wrong with them.’ P3.*

#### The resident perspective

Residents rarely measured blood glucose themselves. According to the physicians, many residents would have been able to do so, but the task was assigned to the nurses. The auxiliary nurses said that they involved the residents in the measurements to some extent, either by assisting those able to do it themselves, or by talking the residents through the process.

*‘Yes, [we’ll say] “this might be a bit sharp”, “ok, now you will feel a little prick”, like that, but then we’re allowed to do the measurement, as some of the residents don’t perform the measurement themselves. Some are allowed to measure themselves, those who are able to of course, yes. They perform the measurement themselves, and they adjust [the insulin] themselves, but you’re with them, observing and double-checking.’ AN3.*

The nurses were concerned that the CBGM sometimes bothered the residents. They nonetheless stated that the residents, even those with dementia, seldom or never expressed concern or objected to measurement. The physicians shared the same experience, reflecting that most residents were used to the routine after living with diabetes for years.

### Professional competence and understanding of roles

#### Training and responsibility

The auxiliary nurses were given CBGM training by the registered nurses, but did not experience this as entirely appropriate. In their experience, the registered nurses had no consistent method of performing CBGM and very seldom received further training after graduating from nursing college. The registered nurses said that training in performing correct CBGM had been given by an external quality improvement programme managed by Norwegian Quality Improvement of Primary Health Care Laboratories (Noklus) [28], but they confirmed that few courses were provided after graduation. They stated that they were expected to acquire and maintain the necessary knowledge about caring for residents with diabetes. The physicians confirmed this. They expected the registered nurses to be able to differentiate between high, normal and low levels of blood glucose, to be knowledgeable about different insulins and antidiabetic medicines and to provide appropriate management of hypoglycaemia. The nurses followed up this responsibility by engaging in self-study and discussing experiences and questions with colleagues.

*‘You look it up if you encounter a challenge while at work. You will go home, look into it, then discuss it with the physician, and then you gain knowledge in that way. Discussing with colleagues, your experiences. That is something you learn from all the time.’ RN3.*

The nurses expressed a wish for mandatory, inter-professional courses to ensure that everyone has the same information and follows the same guidelines. The physicians supported this, and felt that they had a great responsibility to monitor and tailor the training, as it was often them who discovered that it was inadequate. However, they also emphasised the nurses’ responsibility for giving feedback on lacking procedures or insufficient courses, and that responsibility ultimately rested with the employer.

*‘In my experience, it is often very useful to attend [the nurses’] training. (…) There are often totally different approaches for the nurses compared to the physicians, you know. And they often benefit from seeing it from both angles. And my opinion is that it is a joint responsibility, that you as a physician have a great responsibility to oversee the training given at the nursing home, because you work so closely with the staff and the others involved in the training programme.’ P3.*

#### Awareness and assessment of symptoms

The nurses knew which symptoms would call for an additional measurement or would require notification of the physician, also among residents not diagnosed with diabetes. The registered nurses said that they found it easier to spot hypoglycaemia than hyperglycaemia, while the auxiliary nurses admitted that they sometimes found it difficult to distinguish between the symptoms of these conditions. Physicians thought that registered nurses interpreted diabetes symptoms appropriately, but found that they deviated from their set orders for CBGM and insulin injections due to concerns about potential hypoglycaemia. The registered nurses admitted a tendency to perform CBGM more often than the physician had recommended, and that borderline low or high values made them feel uncertain. However, the physicians emphasised that diabetes is a complicated disease and that residents’ symptoms of hypoglycaemia could cover a surprisingly wide spectrum. They further underlined that proper management depended a lot on precise orders and the opportunity to get regular practice or training in these matters.

*‘Maybe if a resident’s blood glucose is low in the morning, but not very low, more borderline low, somewhat under what’s normal for that resident, you start to think “should I inject insulin, should I not inject insulin?”, because that’s not specified anywhere, you know? (…) And most times they need [insulin] anyway. When they have eaten, [the blood glucose level] will become too high if they don’t get [insulin]. But then, OK, you will still stand there assessing these things, so…’ RN2.*

*‘It’s not a diagnosis that’s based on a blood test, it’s a diagnosis based on a clinical assessment. And it’s a surprisingly wide spectrum for, you know, what is the lower [limit], or when do they experience hypoglycaemia? Some will not experience it before their value is around 2 [mmol/l], while others may experience it around 4 [mmol/l], you know?’ P2.*

### Record keeping

#### Single or double documentation? A two-sided argument

The responsible nurse logged all information about the CBGM, e.g. the time, value, site of pricking, units of insulin given, or food intake, in the resident’s records. Some would record the information on paper in the resident’s medical records, then later, preferably the same day, transfer it to the electronic patient records system, where the physician could examine it at any time. The physicians regarded this as unnecessary double documentation. However, to the nurses, the paper sheets, which were easily accessible in the medicine room or trolley on the ward, made it easier to keep an eye out for deviations, both in the residents’ blood glucose levels and each other’s documentation routines.

*‘Strictly speaking, it is double documentation, but we do also have a paper form where we register [the values]; it’s kept in the resident’s kardex. But we also register it in the electronic patient records system that we use. (…) It makes it easier on the physician’s round to be able to access the results from there, but we do register it both places, and that’s also because we need it to be available on the ward, easily accessible, you know? To look back at how [the blood glucose levels] have been earlier.’ RN3.*

#### Official guidelines or common procedures?

None of the participating nurses was aware of any written template or procedure for how to carry out a CBGM. While the auxiliary nurses expressed concern that this led to staff performing CBGM in many different ways, the registered nurses seemed less concerned about this because they felt that they had a good understanding of the practical aspects of CBGM. The nurses were not familiar with any written procedures for how to manage acute glycaemic events. This surprised the physicians, who stated that local authority guidelines for managing hypo- and hyperglycaemia existed and should be well-known.

*‘I believe that they have been given some written guidelines, or teaching or, but yes. That they have them available and can look it up somewhere, but I’d better look into it again.’ P2.*

Despite differences in familiarity with guidelines, common procedures did exist. The registered nurses used the individually set blood glucose limits for residents who needed rapid-acting insulin as guidance, where these existed. However, they stated that orders given by a physician familiar with the resident made them feel much safer than instructions given by an ambulatory physician. In a serious acute event, the physician was always called upon, while smaller deviations in blood glucose and how they had been handled were communicated between shifts and during the physician’s round. The physicians were dependent on this, since no warning would pop up in the electronic system if a resident’s values were deviant. A possible cause was always sought when unexpected symptoms or CBGM results occurred, and the action taken was based on the information available.

*‘Yes, if we’ve taken a blood glucose [measurement] in the morning, you know, then we almost always inform the afternoon shift nurse about the result. Especially if it’s an unusual one, if it’s a low or a high. So that’s part of the verbal report, in addition to it being registered in the medical records.’ RN3.*

## Discussion

### Principal findings

The results from this study indicate that the healthcare professionals tried to provide patient-centred care by minimising strict glycaemic control and excessive CBGM. However, the rationale for CBGM in these nursing homes may be somewhat expanded due to inadequacies in formal policies and training in diabetes care. Hence, the basis for how the healthcare professionals make decisions about care could be skewed towards blood glucose testing rather than clinical assessment. In addition, few opportunities existed for resident empowerment, since residents seldom took part in decisions concerning the management of their own care.

### CBGM – a safety measure or a source of additional worry?

The participants in our study revealed that training in diabetes management was sparse and inconsistent, and the nurses also felt that clear instructions and written procedures were lacking. This sometimes contributed to a feeling of uncertainty and created fear of inducing hypoglycaemia in residents. Hence, CBGM was used to reassure both staff and residents. The participants had also created systems for preventing and managing acute events, including good communication and thorough documentation procedures.

In a focus group study from the UK addressing healthcare professionals’ concerns about diabetes care in care homes and domiciliary care, the participants stated that, even though regular CBGM and detailed communication between shifts are helpful, knowing your patients well is the key to preventing hypoglycaemia [29]. And, as the physicians in our study pointed out, even though the range of values where residents experience hypoglycaemia can be extremely wide, the registered nurses managed acute situations well. This could be due to good knowledge of signs and symptoms and the fact that they were constantly attentive to their patients. However, the nurses would still confirm their suspicions using CBGM.

Similar findings have been reported by Graue et al., who found that nurses working in nursing homes lacked confidence when interpreting and managing changes in residents with diabetes. Here, the authors point to little time to keep up-to-date about diabetes, few resources that could be consulted, and limited support within and between professions as sources of uncertainty [24]. In our study, the nurses did not seem to lack support from their peers or the physician, but there was a lack of systematic training and common procedures. Performing CBGM not ordered by the physician and keeping glucose logs on paper sheets in the residents’ medical records were therefore used to support their clinical assessments. However, borderline glucose values contributed to further uncertainty about how to handle the situation. Even though the physicians stressed that clinical competence is more important than CBGM, they admitted that inadequate instructions and training probably contributed to this practice.

Several studies have observed inappropriate care to be a consequence of deficiencies in guidelines [15, 19, 30] or formal training in diabetes care for healthcare professionals working in long-term care [25, 29]. Accordingly, a need for training in diabetes care has also been pointed out [10, 24, 25, 29, 31], highlighting areas such as which signs and symptoms to look for, recognising when to perform a CBGM and managing hypoglycaemia. Others have emphasised how continued education in diabetes care could enhance the nursing staff’s knowledge, confidence and professional competence, and lead to improved patient outcomes [11, 31–33]. These findings seem to be transferable to our study population.

### The resident – the centre of attention but not part of the team?

Even though the residents’ quality of life was the participants’ main concern, they seldom or never talked about including the resident in decisions about their diabetes care or CBGM. The registered nurses stated that decisions about CBGM were made jointly between them and the physicians, but they never mentioned the resident as part of the team. This was also reflected in the fact that very few residents performed CBGM themselves.

Two recent studies found that, even though healthcare professionals wanted to provide patient-centred care, several barriers existed that made them take a more traditional approach and carry out activities on behalf of the patient [33, 34]. In Huber et al., the nurses described how complications and comorbidities limited older patients’ ability to manage their diabetes care [33]. Asimakopoulou et al. reported that healthcare professionals had the impression that the concept of empowerment was unfamiliar to older patients, and that they regarded making decisions about treatment as the healthcare professionals’ job [34]. This could perhaps explain the situation our participants find themselves in: wanting to empower the residents, but finding that they are neither willing nor able to take this responsibility.

Asimakopoulou et al.’s study also revealed that most healthcare professionals interpreted the term empowerment to mean giving the patients informed choice about their treatment and that meeting biochemical targets was an indicator of successful empowerment [34]. This stands in contrast to the findings of Huang et al., who reported that community-dwelling older adults with diabetes described their goals in global, functional terms, instead of focusing on biomedical goals [35]. This pragmatic view seems to be mirrored by statements made by the healthcare professionals in our study, as they strive to ensure minimal discomfort for the residents, for instance by accepting a slightly raised blood glucose level and attempting to avoid excessive measurements. This sober-minded approach to care could also be part of the reason why the residents seldom or never protested about nursing staff performing CBGM or managing their treatment. However, in a previous study, we found that 60 % of nursing home residents with diabetes had experienced one or several worryingly low CBGM readings, and 46 % had an HbA1c under 7.0 % (53 mmol/mol) [23]. This discrepancy could reflect the possibility that the healthcare professionals in our focus groups are particularly up-to-date about current recommendations for diabetes management. It is also likely, however, that what one strives for in theory may not be so easy to achieve in practice. This could also be true as regards including the resident as part of the team. While the healthcare professionals we interviewed individualised management as best as they could, they did it based on their own preconceptions of what was considered appropriate and seldom seemed to involve the resident. Huang et al. argue that providers’ awareness of how older people define their goals for managing their diabetes should be improved in order to enable better and more individualised plans to be developed [35]. “Patient-centeredness”, placing the patient or the resident at the centre of the consultation, is the very foundation for achieving empowerment, Asimakopoulou et al. states [36]. Identification of the resident’s wishes and capacities for self-care, as well as any concerns and issues related to their diabetes care, should be done on admission to the nursing home and the care plan should be revised on a regular basis [37]. Often residents are hesitant or anxious to express their wishes or needs to nursing staff, as they fear it will be perceived as conflict behaviour and ultimately will have a negative effect on the care they receive. Hence, it is important to ensure the residents that their opinion matters and that conveying your wishes to the nursing home staff will improve rather than reduce quality of care [37]. To offer the resident to take an active role in their own care, through discussing their views on measurement frequency and CBGM results, as well as providing training or guidance in performing CBGM, may be ways to empowerment. Education and empowerment of nursing staff is also vital to further facilitate resident autonomy [37, 38]. Building professional competence and a healthy and positive work culture among nursing staff will help the staff to be more aware of the residents’ needs and enhance nursing care [37, 38]. This requires access to guidelines, opportunity to attend courses and seminars, as well as an open and positive work environment where discussion of care situations is encouraged.

### Strengths and limitations of the study

Keeping the focus groups profession-specific was both a strength and a necessity. The professional hierarchy could have proved limiting for group dynamics in a mixed group, and the different professionals might have felt that they were not given an opportunity to stress what was important to them. Profession-specific groups and the use of open-ended questions help the participants to share what they see as important, in their own language, concepts and framework for understanding the topic [26]. Even though the researchers belong to different professional groups than those interviewed, the systematic analysis method stays true to the participants’ perspectives and phrasing by creating a condensate in the form of an artificial quote. It also validates the findings and interpretations against the original transcripts, and thus helps to preserve the individual context [27].

The greatest limitation of the study is the difficulty we experienced in recruiting nurses. This resulted in a limited sample size in these two focus groups. The goal was to recruit five to eight participants in each group, as recommended by Malterud [39], but this was only achieved for the physician group. We could have attempted to organise additional focus groups to obtain more material, but we found the interaction between participants to be adequate to elucidate our objectives. Our ambition was not to provide an extensive description of every aspect of CBGM practices in nursing homes, but to explore the breadth of experiences and opinions of the different healthcare professionals involved in this aspect of diabetes care. It is likely, however, that we have included healthcare professionals who are most receptive to the topic. According to Malterud, this may not be a disadvantage, since, with respect to external validity, the number of relevant episodes presented in the focus groups is more important than the number of groups or participants [39].

## Conclusion

We found that the aim of protecting the residents’ safety and well-being may be compromised by systematic inadequacies in procedures and training. The participants in our study focused more on the residents’ quality of life than on glycaemic goals and individualised management as best they could. In nursing homes, it may not always be possible or reasonable to let the residents manage their own treatment, but it is still important to evaluate whether they are able to, and wish to, manage their own disease.

As a follow-up of this study, it would be interesting to use quantitative methods to explore what guidelines, procedures and training opportunities exist for diabetes care in Norwegian nursing homes, and how they are being used. Future studies should also investigate the residents’ perspective on self-care in diabetes management, and efforts should be made to include the residents’ wishes and needs in their care plans.
